# M2-like tumor-associated macrophages drive vasculogenic mimicry through amplification of IL-6 expression in glioma cells

**DOI:** 10.18632/oncotarget.13661

**Published:** 2016-11-26

**Authors:** Lin Zhang, Yangyang Xu, Jintang Sun, Weiliang Chen, Lei Zhao, Chao Ma, Qingjie Wang, Jia Sun, Bin Huang, Yun Zhang, Xingang Li, Xun Qu

**Affiliations:** ^1^ Institute of Basic Medical Sciences, Qilu Hospital of Shandong University, Jinan, P.R. China; ^2^ Department of Neurosurgery, Qilu Hospital of Shandong University and Brain Science Research Institute, Shandong University, Jinan, P.R. China; ^3^ Key Laboratory of Cardiovascular Remodeling and Function Research, Qilu Hospital, School of Medicine, Shandong University, Jinan, P.R. China

**Keywords:** macrophages, glioma, vasculogenic mimicry, IL-6, PKC

## Abstract

Vasculogenic mimicry (VM) has offered a new horizon for understanding tumor angiogenesis, but the mechanisms of VM in glioma progression have not been studied explicitly until now. As a significant component of immune infiltration in tumor microenvironment, macrophages have been demonstrated to play an important role in tumor growth and angiogenesis. However, whether macrophages could play a potential key role in glioma VM is still poorly understood. Herein we reported that both VM and CD163^+^ cells were associated with WHO grade and reduced patient survival, and VM channel counting was correlated to the number of infiltrated CD163^+^ cells in glioma specimens. *In vitro* studies of glioma cell lines implicated that M2-like macrophages (M2) promoted glioma VM. We found that conditional medium derived from M2 amplified IL-6 expression in glioma cells. Furthermore, our data indicated that IL-6 could promote glioma VM, as blocking IL-6 with neutralizing antibodies abrogated M2-mediated VM enhancement. In addition, the potent PKC inhibitor bisindolylmaleimide I could prevent M2-induced IL-6 upregulation and further inhibited glioma VM facilitation. Taken together, our results suggested that M2-like macrophages drove glioma VM through amplifying IL-6 secretion in glioma cells via PKC pathway.

## INTRODUCTION

Gliomas are a heterogeneous group of neoplasms and constitute approximately 50% of primary brain tumor [1], the most common being the highly malignant glioblastoma [2]. Despite therapeutic options have improved, the median survival among patients with glioblastoma is only 14.6 months [3]. As glioblastomas are highly vascularized tumors [4], anti-angiogenic therapies targeting endothelial cells have received much attention and investigation [5]. However, the conventional anti-angiogenic therapy, which seemed promising initially, shows transitory and incomplete efficacy [6–9]. These studies indicated that there may be other blood supply forms in tumor tissues. Maniotis *et al*. first demonstrated tumor vasculogenic mimicry (VM), which refers to tumor cells directly lined up to form blood vessels, independent of endothelial cells [10]. Accumulated results have shown that VM exists in various malignant tumors and is linked to resistance to anti-angiogenic therapy and the poor prognosis of cancer patients [11–13]. As an alternative to conventional anti-angiogenic therapy, the identification of molecules and signaling pathways relating to VM may offer potential therapeutic targets to improve treatment [14].

In solid tumors, the course of pathology is controlled not only by the genetic makeup of the tumor cells, but also depends on the interplay with tumor microenvironment [15]. A striking feature of tumor microenvironment is the large number of immune cells, which accumulate in tumor mass and involve in the tumor progression [16]. The most abundant immune cells infiltrating in solid tumor are macrophages, representing up to 50% of all tumor mass [16]. In glioblastoma tumor-associated macrophages (TAMs) constitute up to 30% of tumor mass [17, 18]. Moreover, TAMs infiltrated in glioblastoma tissues have been reported to express the elevated M2 cell markers, CD14, CD68, CD163, and CD204 [19]. Recent work indicated that TAMs infiltration contributed to proangiogenic effects and was associated with resistance to anti-angiogenic therapy in several solid tumors [20–22], including glioblastoma [23]. These findings suggested that targeting TAM-promoting angiogenesis in tumor progression can be a universal method to overcome anti-angiogenic drug resistance.

Another important hallmark of cancer is considerable cytokines and chemokines in tumor microenvironment [24, 25]. Increasing data described that these immune mediators could create a favorable environment to support tumorigenesis by driving the functions of immune cells and tumor cells [26, 27]. One such factor is the pleiotropic cytokine, interleukin-6 (IL-6); a potent mediator that is omnipresent in the inflammatory microenvironment of most solid tumors, including glioma [28]. Although previous investigations have confirmed that IL-6 is important in both physiological and pathological angiogenesis [29, 30], IL-6 has recently received more attention as a critical cytokine implicated in the angiogenesis of several human cancers [31–34]. These investigations indicated that IL-6 could induce the release of VEGF from the tumor cells promoting angiogenesis [31–34]. These studies support the notion that IL-6 may be an important regulatory molecule in tumor angiogenesis, but whether it plays a direct role in the process of glioma VM formation is still vague. In this report, we preliminarily explore a correlation of VM with TAMs in human glioma tissues, and the regulatory mechanisms how M2-like macrophages promote VM through amplifying IL-6 secretion in glioma cells.

## RESULTS

### Correlation of VM level with CD163^+^ TAM infiltration in human glioma tissue

**Figure 1:**
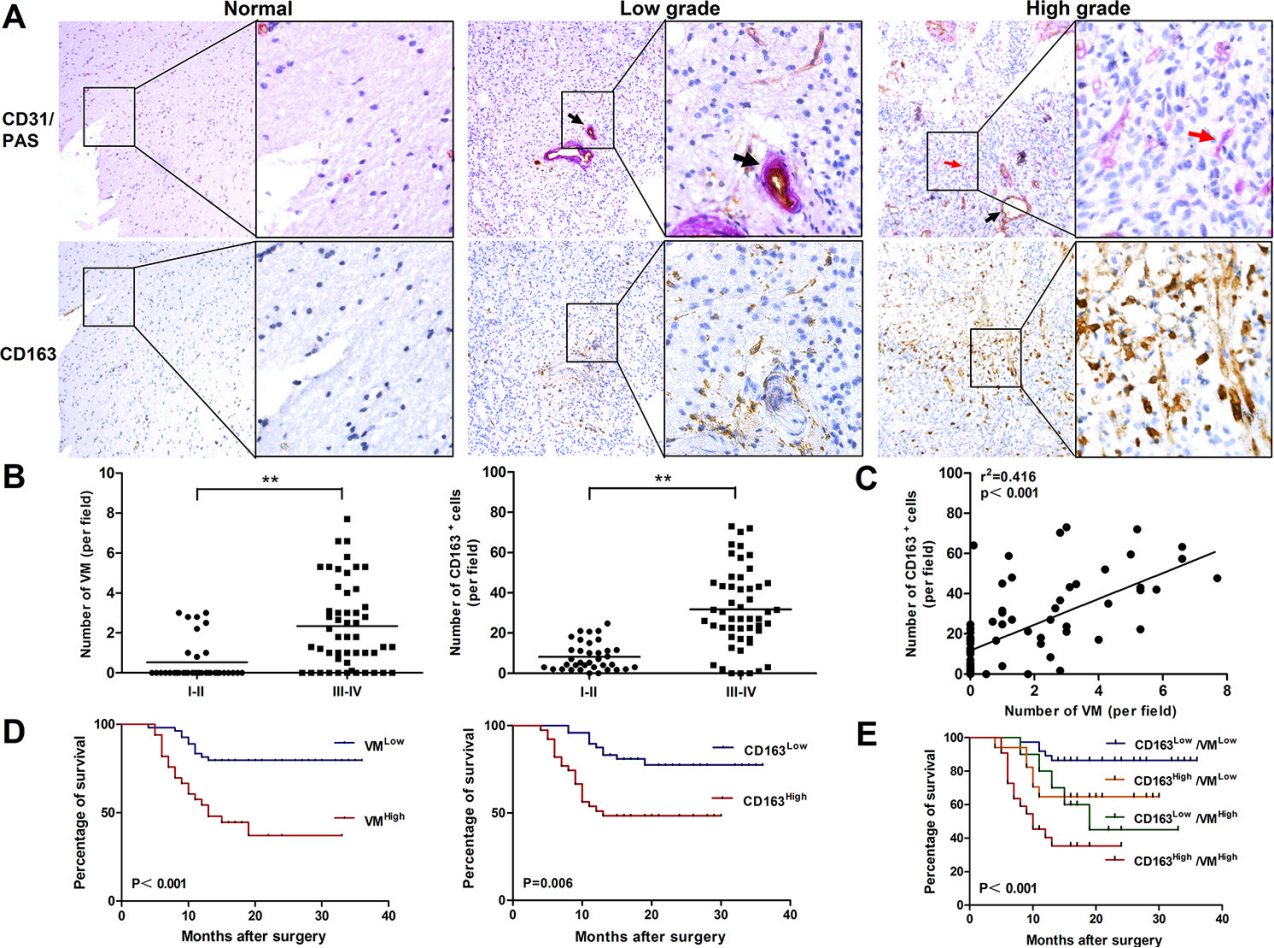
Correlation of VM level with CD163 expression in human glioma tissue and overall survival for glioma patients (**A**) Immunohistochemical staining of CD31/PAS and CD163 in glioma specimens (*n* = 87). Shown were two representative serial sections (small: 100×; large: 400×). Blood vessel (PAS^+^CD31^+^) was shown by black arrow and VM (PAS^+^CD31^−^) was shown by red arrow. (**B**) Numbers of VM and CD163^+^ cells were both correlated with glioma WHO grade. (**C**) The correlation between VM counting and CD163^+^ cell number in each tumor sample. **P < 0.01. (**D**) Both VM (*P <* 0.001) and CD163 (*P* = 0.006) were correlated with overall survival on glioma patients between low and high subgroups. (**E**) Survival analysis was performed between subgroups combined with VM and CD163. Kaplan–Meier method was used to plot survival curves and statistical significance was determined by log-rank test (*P <* 0.001).

To investigate whether the density of M2-like TAMs correlated with the VM level in human glioma, we detected the expression of CD163 (a marker of human M2-like TAMs), CD31 and PAS (markers for vessel) in 87 human glioma specimens by immunochemical staining. As shown in Figure 1A, CD163^+^ cells were present throughout the tumor tissue, and grade IV glioma specimens had the highest number of infiltrated CD163^+^ cells (Figure 1A). We next explored VM in glioma specimens by CD31/PAS dual staining. The VM (CD31^−^PAS^+^) number in high grade was significantly higher compared with low grade or normal brain with edema (Figure 1A, red arrow). As shown in Figure 1B, the levels of VM and infiltrated CD163^+^cells were positively correlated with glioma grade (*P <* 0.01; *P <* 0.01, respectively). More importantly, a correlation of CD31^−^PAS^+^ vessels and CD163^+^ cells was observed in glioma and normal brain tissues. As shown in Figure 1C, VM level is closely associated with CD163^+^ TAM infiltration (r^2^ = 0.416, *P* < 0.001). Taken together, these results clearly revealed a significant positive correlation between VM level and CD163^+^ TAM infiltration in human glioma tissues.

### VM level and CD163 density are associated with survival and clinicopathologic parameters of glioma patients

To determine whether there were prognostically significant association between VM, or CD163 and patient survival, Kaplan-Meier survival curves were then plotted. As shown in Figure 1D, CD163 low group had a significant survival advantage compared with CD163 high group (*P* = 0.006), and this survival advantage was also shown in VM low group (*P <* 0.001). We further analyzed patient survival with the combination of CD163 with VM. More significant value was observed in overall patient survival than one single factor (*P <* 0.001, Figure 1E). The association between VM numbers, CD163^+^ TAM counts and the clinicopathological status of patients with glioma was then analyzed (Table 1). The number of VM and CD163^+^ TAMs increased with aggressive tumor biology defined by advanced WHO grade (*P <* 0.001). VM channels increased with higher tumor burden as defined by tumor size (*P* < 0.05). No correlation was observed in gender, age, tumor location, KPS and growth pattern of tumor. These data suggested that VM level and the density of CD163^+^ TAMs were associated with the progression of human glioma.

**Table 1:** Relationship between expression of CD163, VM and clinicopathological parameters

Parameters	CD163 expression		*P*	VM expression		*P*
	Low High	Low High		Low High	Low High	
Gender			0.623			0.238
Male	27	24		29	22	
Female	21	15		25	11	
Age			0.166			0.307
> 50	14	17		17	14	
≤ 50	34	22		37	19	
KPS			0.104			0.119
≥ 80	44	31		49	26	
< 80	4	8		5	7	
Location			0.074			0.239
Frontal	14	6		11	9	
Temporal	11	8		13	6	
Parietal	14	6		11	9	
Occipital	5	7		7	5	
> 1 lobe	4	12		12	4	
Tumor size (diameter)			0.252			0.027
> 5cm	14	16		12	18	
≤ 5cm	34	23		42	15	
WHO grade			<0.001			<0.001
I	8	0		8	0	
II	27	1		22	6	
III	3	15		9	9	
IV	10	23		15	18	
Surgery			0.051			0.302
Gross total resection	48	36		53	31	
Partial resection	0	3		1	2	

### M2-like macrophages drive VM formation of glioma cells *in vitro*

**Figure 2:**
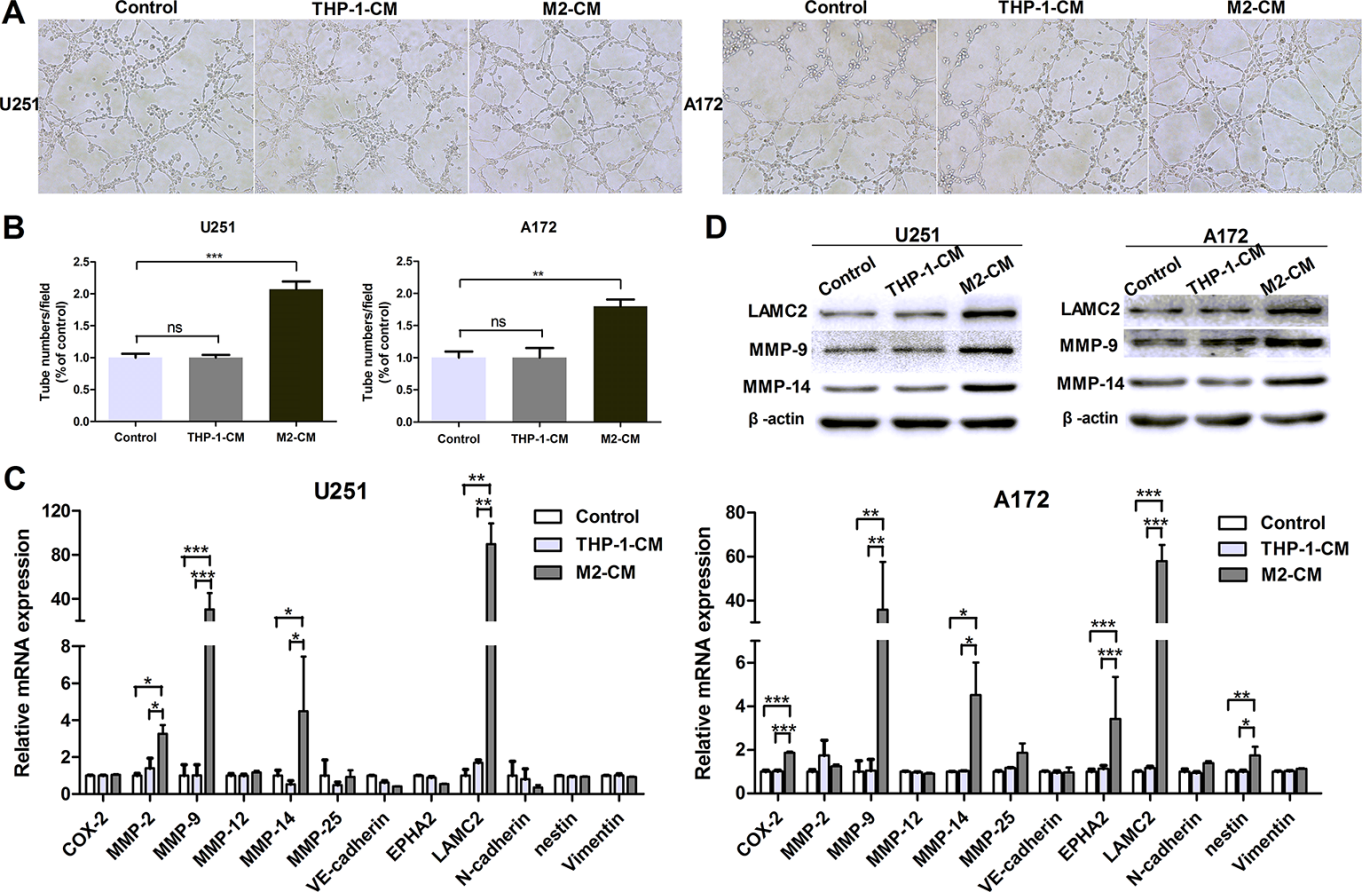
M2-like macrophages drive VM formation of glioma cells *in vitro* The glioma cells were incubated in the conditioned medium (CM) from THP-1 or M2 cells for 24 h. The glioma cells cultured in DMEM medium were used as control. (**A**, **B**) Representative images (A) and quantification (B) of tubule formation assay on Matrigel matrix *in vitro* (×100). (**C**) qRT-PCR was performed to evaluate the effect of THP-1-CM or M2-CM on VM markers. (**D**) Western blotting was used to detect the expression of VM associated proteins, including MMP-9, MMP-14 and LAMC2. Each bar represents the mean ± SEM (*n* = 3). *P < 0.05; **P < 0.01; ***P < 0.001; ns, no significance.

M2-like macrophages was induced by THP-1 cells *in vitro*. THP-1-derived M2-like macrophages expressed high levels of M2 type markers (CD163, CD206, IL-10, IL-1RA and CCL17) and low levels of M1 type markers (TNF-a, IL-1β and IL-12) (Supplementary Figure S1). To determine the effect of M2-like macrophages on VM formation, tubule formation assays were performed. As shown in Figure 2A and 2B, treatment by conditioned medium from M2-like macrophages (M2-CM) increased approximately 2 fold changes of the tubule structures in U251 cells, compared to THP-1-CM-treated group or control group (*P <* 0.001, *P <* 0.001, Figure 2A and 2B). This advantage of the number of tubes was also shown in M2-CM-treated A172 cells (*P <* 0.01, *P <* 0.01, Figure 2A and 2B). While there were no significant differences between group cocultured with THP-1-CM and control group in both cells (*P* > 0.05; *P* > 0.05, Figure 2A and 2B). The results indicated that M2-like macrophages enhanced the tube formation abilities of glioma cells.

To further examine the roles of M2-like macrophages in VM and to explore the potential mechanisms, we studied VM-related molecular markers previously identified [35]. As shown in Figure 2C, the transcriptional levels of VM markers MMP-9, MMP-14 and LAMC2 were significantly up-regulated after coculture with M2-CM in U251 and A172 cells, while they were not significantly altered in THP-1-CM-treated group (Figure 2C). This upregulation was also verified by western blotting (Figure 2D). Therefore, our data showed that M2-like macrophages promoted the VM formation in glioma cells.

### M2-like macrophages amplify IL-6 expression in glioma cells

**Figure 3:**
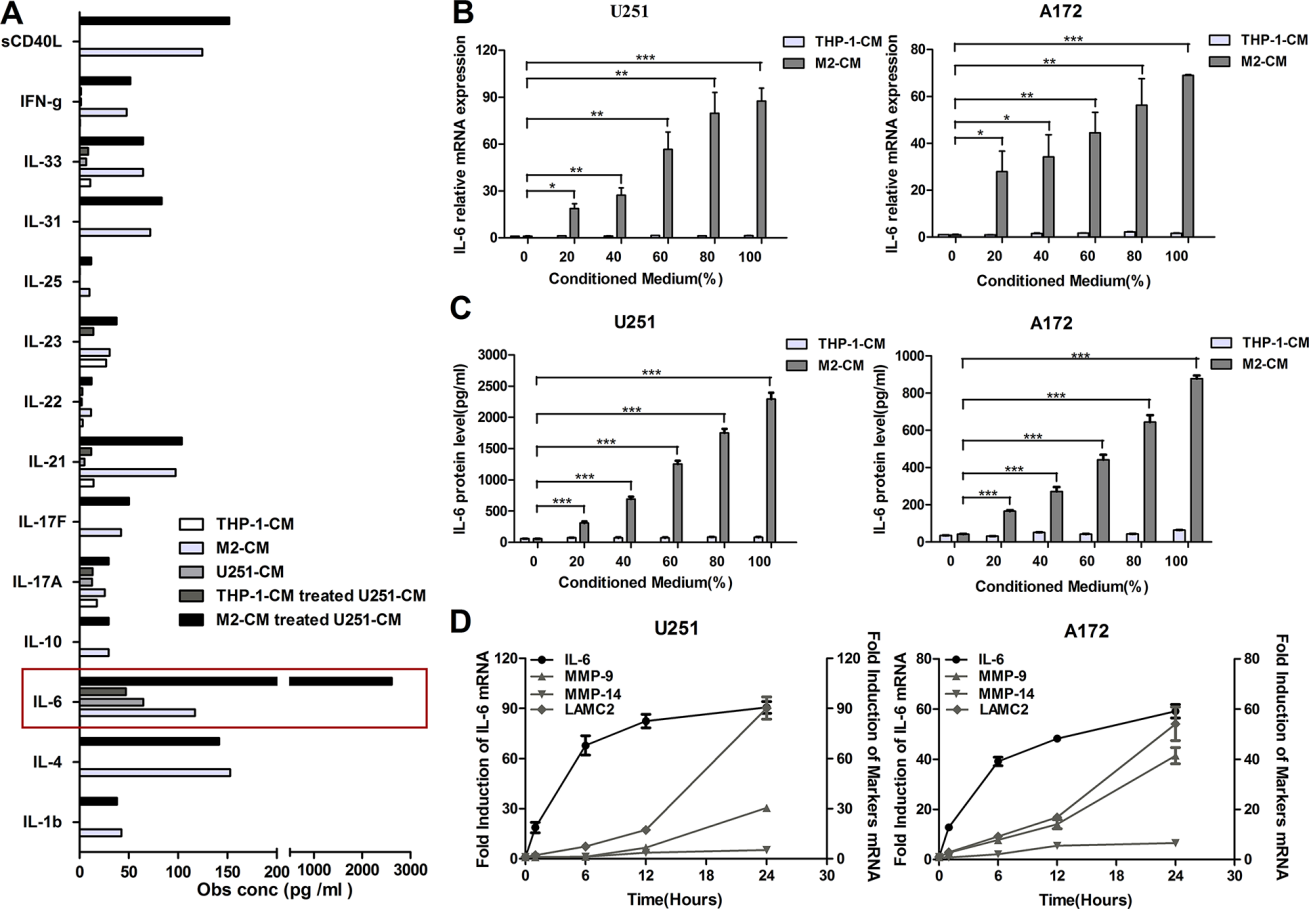
M2-like macrophages amplify IL-6 expression in glioma cells (**A**) The Bio-Plex Pro^TM^ Human Th17 Cytokine Assay were used to detect the cytokines in the supernatant of THP-1, M2, U251, THP-1-CM/U251 coculture system and M2-CM/U251 coculture system. (**B**, **C**) Dose-dependent induction of IL-6 expression in human glioma cells by M2-CM for 24 h. The level of IL-6 mRNA was determined by qRT-PCR (B) and IL-6 concentration in the medium was detected using ELISA assay (C). (**D**) Time course of IL-6 and VM marker mRNA expression in glioma cells stimulated with M2-CM. Transcript levels were measured at indicated time after stimulation using qRT-PCR. Each bar represents the mean ± SEM (*n* = 3). *P < 0.05; **P < 0.01; ***P < 0.001.

In the above study, we demonstrated that M2-like macrophages promoted glioma VM. However, the mechanism by which M2-like macrophages promoted glioma VM is still unclear. Therefore, in order to explore the potential target factor and screen the responsible cytokine, cytokine array was performed to analyze conditioned medium from THP-1, M2, U251, THP-1-CM/U251 coculture system and M2-CM/U251 coculture system (Figure 3A). Interestingly, IL-6 production by glioma cells was dramatically increased in the medium of M2-CM/U251 coculture system, compared to other groups (Figure 3A). The upregulation of IL-6 was further confirmed by qRT-PCR and ELISA in U251 cells. To generalize our finding, IL-6 upregulation was also examined in A172 cells. As shown in Figure 3B and 3C, compared to THP-1-CM-treated group, transcription and secretion of IL-6 in glioma cells were markedly increased by M2-CM in dose-dependent manner.

To determine the relative timing of IL-6 and VM marker expression following stimulation, mRNA levels of IL-6 and VM markers in glioma cells stimulated with M2-CM were evaluated by qRT-PCR at indicated time points (Figure 3D). IL-6 transcript levels rose first, reaching near-peak expression at 6 hours, whereas VM marker expression lagged behind, rising steadily over 24 hours.

### IL-6 upregulation is responsible for VM promotion in glioma cells

**Figure 4:**
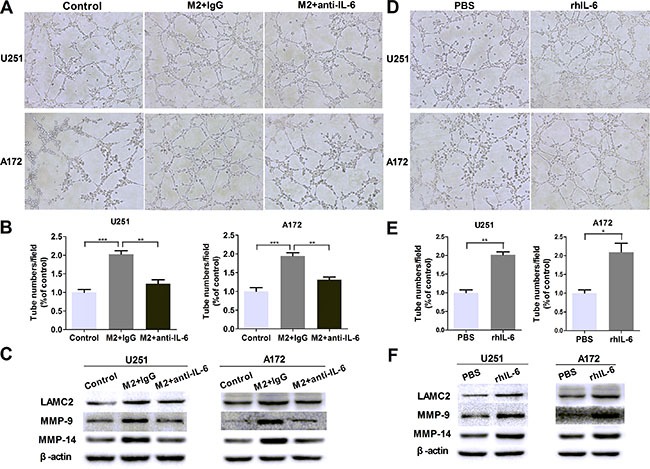
IL-6 upregulation is responsible for VM promotion in glioma cells (**A**, **B**) Glioma cells were treated with DMEM medium or M2-CM in the presence or absence of anti-IL-6 at 1 μg/ml, or isotype-matched IgG (IgG) control for 24 hours. Representative images (A) and quantification (B) of tubule formation assay on Matrigel matrix *in vitro* (×100). (**C**) As previously treatment, the protein levels of VM markers were then detected by Western blotting. (**D**, **E**) Glioma cells were treated with rhIL-6 in a concentration of 100 ng/mL or PBS control for 24 hours. Representative images (D) and quantification (E) of tubule formation assay on Matrigel matrix *in vitro* (×100). (**F**) VM markers protein of glioma cells, alone or after cultured with rhIL-6. Each bar represents the mean ± SEM (*n* = 3). *P < 0.05; **P < 0.01; ***P < 0.001.

In order to determine whether there was a correlation of IL-6 upregulation with VM enhancement in glioma cells, IL-6 neutralizing antibody (anti-IL-6, 1 μg/ml) was used to investigate the effect of IL-6. As shown in Figure 4A and 4B, the number of tubes was significantly decreased after adding anti-IL-6 into the coculture system, and the protein levels of VM markers were abated as well (Figure 4C). In addition, recombinant human IL-6 (rhIL-6, 100 ng/ml) was employed to measure the effect of IL-6. As shown in Figure 4D and 4E, the number of tubes was increased by using rhIL-6 alone compared with their corresponding controls (*P <* 0.01, *P <* 0.05), and VM marker expression was augmented correspondingly (Figure 4F). Taken together, these results indicated that IL-6 may play a significant role in M2-CM-enhanced VM in glioma cells.

### M2-like macrophages promote IL-6 and VM in glioma cells via PKC pathway

**Figure 5:**
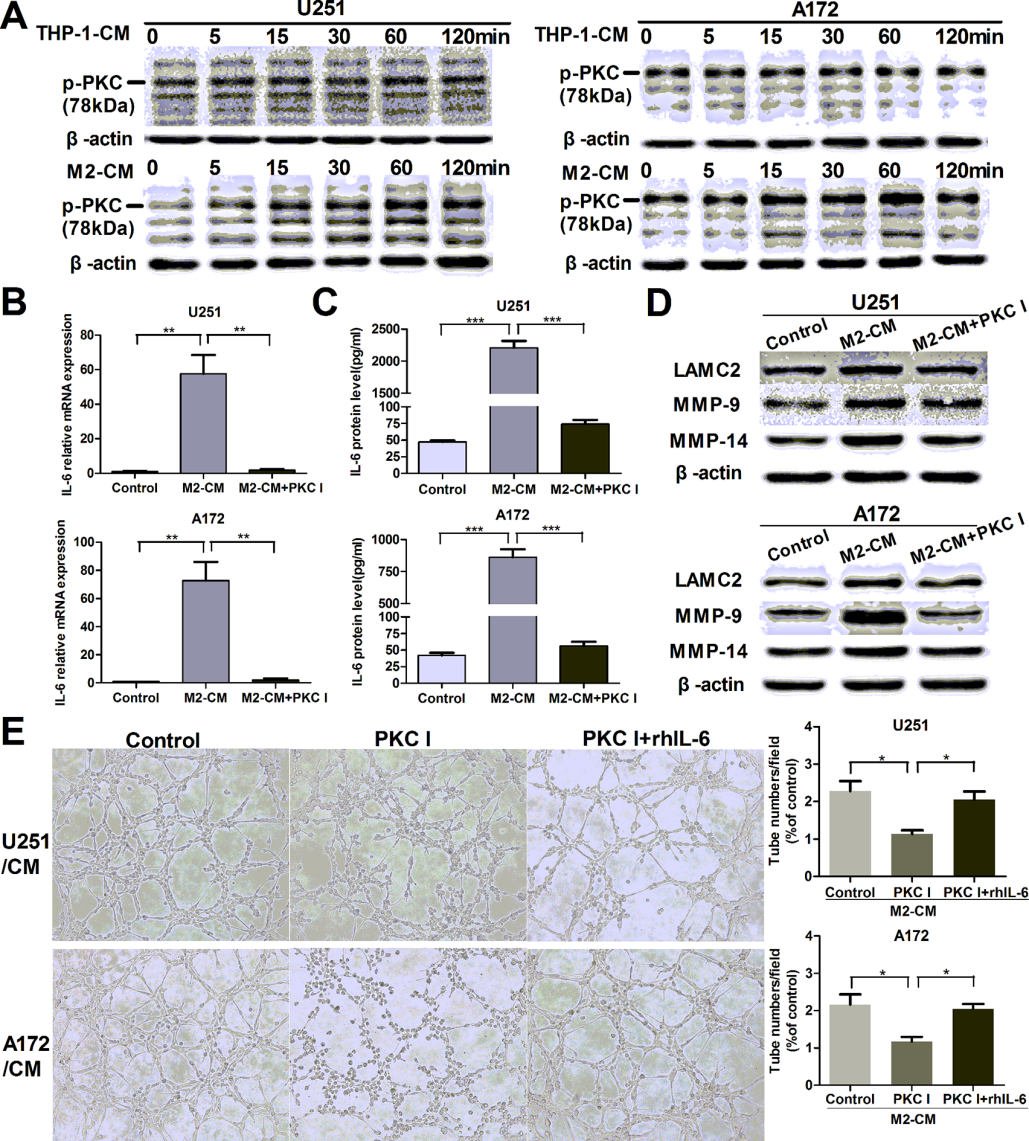
M2-enhanced IL-6 and VM in glioma cells via PKC pathway (**A**) Glioma cells were incubated with THP-1-CM or M2-CM for indicated time, the phosphorylation of PKC (p-PKC, Pan) was determined by Western blotting. (**B**, **C**) Glioma cells were incubated with DMEM medium, M2-CM or M2-CM containing Bisindolylmaleimide I (PKC I) for 24 h, IL-6 transcription (B) and concentration (C) in CM were determined by qRT-PCR and ELISA respectively. (**D**) As previously treatment, the protein levels of VM markers were then detected by Western blotting. (**E**) Representative images and quantification of tubule formation assay in glioma cells incubated in M2-CM, M2-CM containing Bisindolylmaleimide I or M2-CM containing Bisindolylmaleimide I with rhIL-6 for 24 h (×100). Each bar represents the mean ± SEM (*n* = 3). *P < 0.05; **P < 0.01; ***P < 0.001.

We went on to investigate the mechanisms in the process of M2-mediated VM promotion. It has been reported that PKC signal pathway played a key role in IL-6 production [36]. To determine whether PKC pathway was involved in our study, we measured the phosphorylation of PKC pathway treated with CM for indicated time points. As shown in Figure 5A, glioma cells were stimulated with M2-CM at different time, and a transient upregulated phosphorylation of PKC pathway was measured by Western blotting. PKC inhibitor Bisindolylmaleimide I significantly inhibited IL-6 transcription (Figure 5B) and secretion (Figure 5C) induced by M2-CM. In addition, VM markers (Figure 5D) and VM formation (Figure 5E) were also inhibited by Bisindolylmaleimide I. However, rhIL-6 significantly rescued the inhibiting effect of Bisindolylmaleimide I on VM in glioma cells cocultured with M2-CM (Figure 5E). In addition, inhibitor of p38 MAP kinase (SB 203580) and inhibitor of phosphatidylinositol 3-kinase (Wortmannin) were also used to examine their effects on IL-6 production, while their significant inhibitory effects were not observed in glioma cells (data not shown). In all, these results indicated that the potent PKC inhibitor bisindolylmaleimide I could prevent M2-induced IL-6 upregulation and further inhibit tubule formation of glioma cells.

### Monocyte-derived M2 macrophages also induce VM promotion via IL-6 amplification in glioma cells

**Figure 6:**
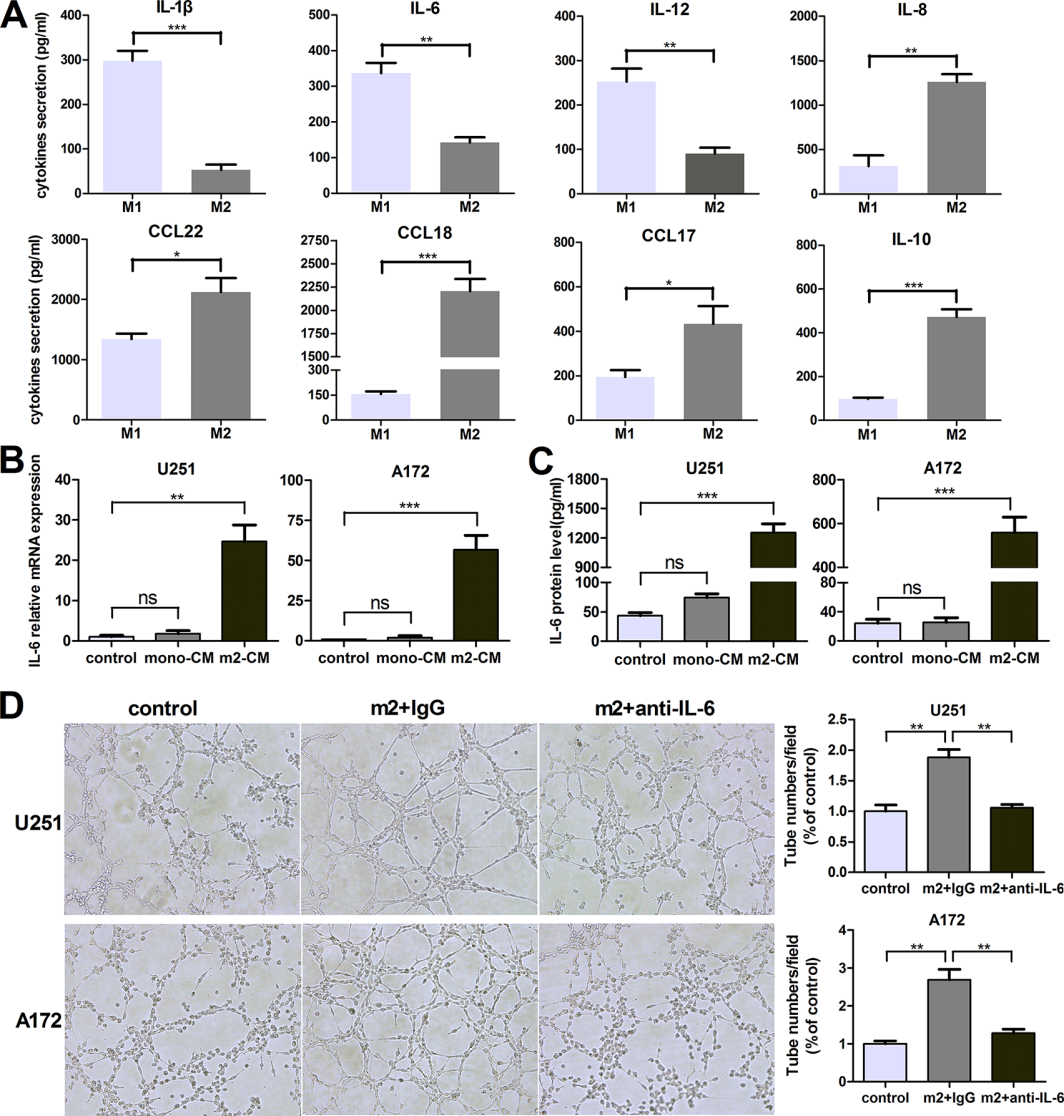
Monocyte-derived M2 macrophages also induce VM promotion via IL-6 amplification in glioma cells (**A**) The cytokines/chemokines with different concentration secreted by human peripheral blood monocyte-derived M1/M2 macrophages were evaluated by ELISA kit. (**B**, **C**) Glioma cells were incubated with DMEM medium, mono-CM or m2-CM for 24 h, IL-6 transcription (B) and concentration (C) in CM were determined by qRT-PCR and ELISA, respectively. (**D**) Glioma cells were treated with DMEM medium or m2-CM in the presence or absence of anti-IL-6 at 1 μg/ml, or isotype-matched IgG (IgG) control for 24 hours. Representative images and quantification of tubule formation assay on Matrigel matrix *in vitro* (×100). Each bar represents the mean ± SEM (*n* = 3). *P<0.05; **P < 0.01; ***P < 0.001; ns, no significance. mono-CM: conditioned medium from CD14^+^ monocytes. m2-CM: conditioned medium from monocyte-derived M2 macrophages.

To generalize our findings, we isolated monocytes from human peripheral blood mononuclear cells by magnetic cell sorting using CD14 microbeads. Monocyte-derived M2 macrophages expressed high levels of M2 type markers (CD163, CD206, CCL18 and CCL17) and low levels of M1 type markers (TNF-a, IL-1β, IL-6 and IL-12) (Supplementary Figure S2). As shown in Figure 6A, monocyte-derived M2 macrophages secreted high levels of M2 type cytokines/chemokines (CCL22, CCL18, CCL17 and IL-10) and low levels of M1 type cytokines (IL1-β, IL-6, IL-12 and IL-8).

In order to determine whether monocyte-derived M2 macrophages could promote VM through IL-6 upregulation in glioma cells, IL-6 transcription and concentration were determined by qRT-PCR and ELISA. As shown in Figure 6B and 6C, IL-6 production by glioma cells was dramatically increased in the medium of monocyte-derived M2-CM/glioma coculture system, compared to other groups. And then IL-6 neutralizing antibody (anti-IL-6, 1 μg/ml) was used to investigate the effect of IL-6. As shown in Figure 6D, the number of tubes was significantly increased after treated by monocyte-derived M2-CM compared to control group (*P <* 0.01), but the increments were abrogated by anti-IL-6 (*P <* 0.01). Taken together, monocyte-derived M2 macrophages also induced VM promotion via IL-6 amplification in glioma cells.

## DISCUSSION

Glioblastoma is recognized as a highly angiogenic malignant brain tumor [4]. However, conventional anti-VEGF agents have not significantly extended the life expectancies of glioblastoma patients [6]. The discovery of VM has offered a new horizon for understanding tumor angiogenesis. Meanwhile, it should be noticed the complexity and diversity of the VM-related signal transduction events, including VE-cadherin, EPHA2, Twist 1, COX-2 [37]. However, the molecular mechanisms of glioma VM formation have not been studied explicitly until now. Therefore, in the present study we preliminarily explored the possible mechanisms of this process and provided additional information. Our results showed that both VM and CD163^+^ TAMs were associated with WHO grade and reduced patient survival, which were consistent with previous studies [13, 19]. After analysis of relevance, we found there was a closely positive correlation between VM level and CD163^+^ TAMs infiltration, which is a significant phenomenon and implies TAMs may involve in VM formation of glioma (Figure 1).

Recently, accumulating experiments have highlighted the critical role of TAMs during tumor angiogenesis. Previous study confirmed that recurrent glioblastomas showed an increased TAMs infiltration after anti-angiogenic therapy [23], and TAMs promoted angiogenesis in glioma by upregulating the expression of VEGF and pro-inflammatory cytokines that prevented normal vessel formation [38]. VM as an alternative to angiogenesis, hence, glioma VM formation might be influenced by TAMs as well. Moreover, it has been reported that glioma-associated macrophages expressed the elevated M2 cell markers [19]. In order to further examine the effect of TAMs on VM formation, THP-1-derived M2-polarized macrophages (M2-like macrophages) and monocyte-derived M2 macrophages were used as a macrophage model which had a M2 functional profile [39–41]. Data obtained from our study indicated that conditioned medium from M2 cells (M2-CM) increased the tubule formation number in glioma cells, as well as VM marker expression, including LAMC2, MMP-9 and MMP-14 (Figure 2). Previous results have shown that Bevacizumab accelerated metastasis in models of ovarian cancer, with marked VM formation in mice receiving short-term therapy [42]. Therefore, our finding of M2-mediated glioma VM facilitation may be the potential key factor for resistance to conventional anti-angiogenic therapy of glioma.

We further explored possible molecular mechanisms that may be responsible for the proangiogenic effect of M2-like macrophages. Although activated myeloid cells can directly secrete proangiogenic factors [43, 44], TAMs may also regulate tumor VM in an indirect way. Interestingly, in the current study, cytokine array screened the potential key factor IL-6 which was the most significantly upregulated cytokine in glioma cells after stimulation by M2-CM (Figure 3). Recently, a growing number of publications showed that IL-6 was a key player in chemoresistance in various types of malignant states. A study conducted by Conzeet *et al*. showed that IL-6 and its downstream signaling have been found to be responsible for multidrug resistance [45]. A phase II study suggested that the extent of IL-6 increase in the plasma during treatment was associated with an inferior outcome in patients with rectal and ovarian cancer after bevacizumab and chemoradiation treatment, and an inferior outcome in patients with advanced HCC after sunitinib therapy [46, 47]. Besides, it has been demonstrated that IL-6 promoted VEGF-induced glioma angiogenesis [48, 49]. These results implied that IL-6 may be associated with the resistance to anti-angiogenic therapy in cancer, including glioma. Although the cytokine assay performed in this study did not include all the angiogenic factors secreted by glioma cells, these results in our present study have indicated that M2-CM induced VM enhancement, at least partly via the IL-6 amplification in glioma cells. These findings indicated another relevant mechanism of resistance to anti-angiogenic therapy and may aid in the identification of new targets for combinatorial antiangiogenic therapy.

The M2-CM facilitated the IL-6 production and VM formation of glioma cells via PKC pathway compared to THP-1-CM, and the facilitation was mostly abrogated by inhibition of PKC signaling (Figure 5). The results indicated that the potent PKC inhibitor could prevent IL-6 upregulation and further destroy tubule formation of glioma cells. PKC is a family of phospholipid dependent serine/threonine kinases that function in numerous different cell types. In astrocytes, PKC was previously shown to be important for IL-6 induction [36]. Since PKC activation can be a very fast process, sometimes disappearing already after a few minutes [50], and PKC could be invoked in our experimental system ranged from approximately 5 min to 3 h, it became virtually impossible to identify the responsible PKC isoforms. In view of the complexity of the PKC pathway, the specific mechanisms which M2-CM promoted IL-6 expression and VM formation in glioma cells need further investigation by isoform-specific inhibitors.

**Figure 7:**
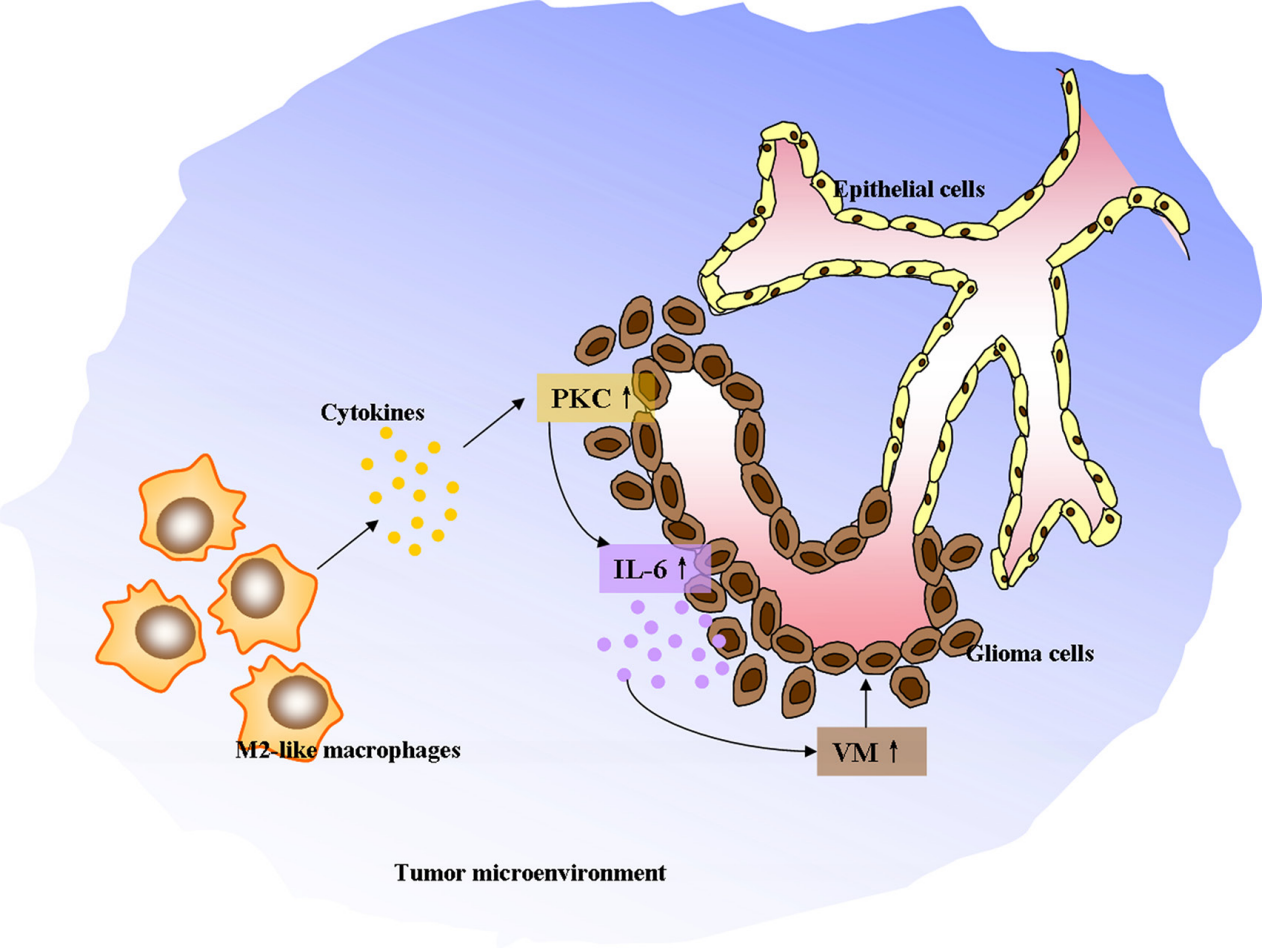
The schematic diagram depicting M2-like tumor-associated macrophages driving VM formation through amplification of IL-6 expression in glioma cells via PKC signaling

In summary, these data indicated that VM level was positively correlated with the number of infiltrated CD163^+^ M2-like TAMs in glioma specimens. The *in vitro* studies suggested that M2-like macrophages and monocyte-derived M2 macrophages drove VM formation through upregulating IL-6 expression in glioma cells via PKC pathway. A schematic diagram was made to depict the detailed mechanisms (Figure 7). Nevertheless, other angiogenic and anti-angiogenic peptides produced by TAMs and glioma cells may also contribute to tumor angiogenesis via different signaling pathways. Thus, further investigations are needed to define the optimal targets for treatment of glioma patients.

## MATERIALS AND METHODS

### Patients and samples

Tissue samples from 87 patients diagnosed with glioma were obtained during the surgical tumor resection at the Department of Neurosurgery, Qilu Hospital of Shandong University. None of the patients had received preoperative adjuvant therapy and KPS score was performed before surgery. The glioma specimens were classified according to the WHO classification criteria and verified by two experienced pathologists. The four normal brain tissues were collected from patients with hernia during surgical decompression. This study was in accordance with the Declaration of Helsinki, and written informed consent was obtained from each patient. The Ethics Committees of Qilu Hospital of Shandong University approved the experiments.

### Reagents and antibodies

Isotype-IgG, rhIL-4, rhIL-13, M-CSF, rhIL-6, anti-IL-6 and human IL-6 Quantikine ELISA were purchased from R&D Systems. Phorbol 12-myristate 13-acetate (PMA) was purchased from Sigma. Bisindolylmaleimide I (protein kinase C (PKC) inhibitor) was purchased from Calbiochem. Matrigel was from BD Biosciences. The primary human antibodies in this study were used: mouse anti-CD163, rabbit anti-CD31 (ZSGB-BIO); rabbit anti-MMP14, rabbit anti-LAMC2 (Abcam); rabbit anti-MMP9, rabbit anti-Phosphorylated PKC (pan) (Cell Signaling Technology); mouse anti-β-actin (Beyotime).

### Immunohistochemical staining

Paraffin sections (4-μm thick) of glioma tissue specimens were prepared for immunohistochemistry. Briefly, the sections were deparaffinized, rehydrated, boiled in 1 mM EDTA (pH 8.0) for antigen retrieval and blocked with goat serum for endogenous peroxidase activity to reduce nonspecific staining. After the incubation with primary antibodies against CD163 at 4°C overnight, the specimens were then incubated with poly-HRP secondary antibodies for 30 min. Finaly, the sections were stained with diaminobenzidine and the nuclei were counterstained with hematoxylin. Images were captured using an Olympus IX81 microscope.

The specimens were assessed by two independent pathologists without knowing any clinicopathologic variables. For CD163 evaluation in glioma samples, the five representative areas (hot spots) were identified by scanning the whole tumor section at low power, followed by counting positive staining cells in each hot spot at 400× magnification, and the mean number per section was obtained. Then the mean number of CD163^+^ cells infiltrated in glioma samples was calculated (21.9 per field). A mean number < 22 was considered low, a mean number ≥ 22 was considered high.

### CD31/Periodic acid-Schiff doublestaining

After immunohistochemical analysis of sections for CD31 expression, the sections were exposed to 1% sodium periodate for 10 min, rinsed for 5 min with distilled water, and then incubated for 15 min with PAS in dark at 37°C. The sections were counterstained with hematoxylin and observed using a microscope. CD31 positive vessels indicated blood vessels in tissues, and CD31 negative, PAS positive vessels were defined as VM. We evaluated VM expression in tumor specimens according to the previous protocols. The CD31/PAS dual-staining sections were viewed at 400× magnification using an Olympus IX81 microscope. The mean VM count of five hot spots was calculated as the VM density (VMD) for each section. The average VMD from all glioma sections was calculated (1.5 per field). All cases with the number < 1.5 per field were considered low, the number ≥ 1.5 considered high.

### Cell culture and treatments

Human malignant glioma cell lines U251, A172 and human monocytic cell line THP-1 were obtained from the Cell Bank of Type Culture Collection of Chinese Academy of Science (CBTCCCAS; Shanghai, China). The cell lines had been recently authenticated based on DNA fingerprinting, isozyme detection and cross species checks. All cells were maintained in suspension in Dulbecco's Modified Eagle Medium (DMEM) supplemented with 10% fetal bovine serum (FBS) at 37°C in a humidified incubator with 95% air, 5% CO2.

To generate THP-1-derived M2-polarized macrophages (M2 cells), THP-1 cells (in a six-well plate, 1 × 10^6^ cells/well) were treated with 100 ng/ml PMA for 6 h, and then cultured with PMA plus 20 ng/ml IL-4 and 20 ng/ml IL-13 for another 18 h. For production of the conditioned medium from M2 cells (M2-CM), all PMA, IL-4 and IL-13 were removed by a thorough wash, and M2 cells were further cultured in 2 ml fresh serum-free DMEM medium for another 24 h. In order to prepare conditioned medium from THP-1 cells (THP-1-CM), THP-1 cells were cultured in 2 ml fresh medium for 24 h. Then the conditioned medium were collected respectively and stored at −80°C.

Human peripheral blood monocytes (PBMs) from healthy donors were isolated by density-gradient centrifugation using Ficoll-Hypaque (Pharmacia, Peapack). Monocytes were isolated using anti-CD14 microbeads (Miltenyi Biotec), and more than 97% of the cells acquired were CD14^+^ monocytes as determined by FACS analysis (data not shown). For *in-vitro* monocyte-derived M2 macrophages activation, CD14^+^ monocytes at 1 × 10^6^ cells/mL were treated for 5 days with 100 ng/ml recombinant human macrophage colony-stimulating factor, followed by 20 ng/ml IL-4 and 20 ng/ml IL-13 for 3 days. This study was approved by the institutional review board of Qilu Hospital of Shandong University. Informed consent was obtained from each subject.

### Tubule formation assay for VM

VM was evaluated using a three-dimensional (3D) culture model containing growth factor-reduced Matrigel (BD Biosciences). Briefly, a volume of 50 μL growth factor-reduced Matrigel was plated in 96-well plates, and allowed to polymerize at 37°C for 30 minutes. Next, 2 × 10^4^ cells were trypsinized and resuspended with serum-free medium, and then seeded onto the Matrigel layer. After 9 h incubation at 37°C with 5% CO2, each well was captured directly by an Olympus BX61 fluorescence microscope under a phase-contrast microscope (×100). The images were quantified for the mean area value of randomly selected vascular network meshes by ImageJ software. Each experiment was performed in triplicate.

### RNA extraction and quantitative real-time PCR

Total RNA was extracted from cell lines using Trizol reagent (Invitrogen), and reverse transcriptase reactions were carried out by ReverTra Ace qPCR RT Kit (Toyobo) method in accordance with the manufacturer's instructions to generate cDNA. Quantitative real-time PCR (qRT-PCR) was performed using a SYBR Green Master Mix kit (Toyobo) on the LightCycler 2.0 instrument (Roche Applied Science). mRNA levels were normalized to GAPDH. The primer sequences used in this study were listed in Supplementary Table S1. The absolute expression levels were calculated as concentration ratios using a Roche LightCycler^®^ 2.0 system.

### Western blotting

Total protein was extracted from cells using RIPA buffer containing 1% phenylmethylsulfonyl fluoride (PMSF). The protein concentration in the medium was determined using a BCA Protein Assay Kit (Beyotime). Equal amounts of total protein were subjected to electrophoresis in 10% SDS-polyacrylamide gels and then transferred onto polyvinylidene difluoride membranes. The membranes were blocked by 5% skim milk blocking buffer for 1 hour. Next, blots were incubated with the primary antibody against MMP-9 (1:1,000; CST), LAMC2 (1:1,000; CST), pPKC (1:1,000; CST), β-actin (1:1,000; Beyotime), or MMP-14 (1:1,000; Abcam). The membranes were then incubated with horseradish peroxidase-linked secondary anti-rabbit or anti-mouse antibodies (1:5,000; Beyotime). Finally, the protein bands were visualized using enhanced chemiluminescence (ECL) assay (Thermo).

### Cytokine array

Cytokine array was used to detect the level of cytokines or chemokines in the medium of glioma cells with or without treatment. Glioma cells were seeded in 6-well plates at a density of 2 × 10^5^ cells per well. After incubation for 24 hours, cells were washed with PBS three times and cultured in fresh serum-free medium, THP-1-CM and M2-CM, respectively. After 24 h, the conditioned medium was collected and centrifuged to remove cell debris, and the mediums were frozen at −80°C until further analysis by cytokine array assay. The Bio-Plex Pro^TM^ Human Th17 Cytokine Assay was used in this study in accordance with the manufacturer's instructions.

### Enzymes linked immunosorbent assay (ELISA)

IL-1β, IL-10, IL-12, IL-8, CCL22, CCL18, CCL17 and IL-6 levels in culture mediums were measured using the human ELISA Kit (R&D Systems) in accordance with the manufacture's instruction.

### Statistical analysis

All statistical analysis and experimental graphs were performed using SPSS 13.0 (SPSS, IL, USA) and GraphPad Prism 5 (GraphPad, CA, USA). The relationship between CD163 and VM was examined by Spearman analysis, and the differences of clinicopathological variables were analyzed with χ2-test. Statistical comparisons among groups were made by Student's *t* test and ANOVA. Data were shown as the mean ± standard error of the mean (SEM), and all experiments were repeated independently at least three times. *P* < 0.05 was considered statistically significant.

## SUPPLEMENTARY MATERIALS


