# Supplementary material for: M2-like tumor-associated macrophages drive vasculogenic mimicry through amplification of IL-6 expression in glioma cells
Source: Oncotarget. 2016 Nov 26;8(1):819–32. doi: 10.18632/oncotarget.13661 (PMC5352199; doi:10.18632/oncotarget.13661)
Supplement: Supplementary file 1 [file oncotarget-08-819-s001.pdf]

M2-like tumor-associated macrophages drive vasculogenic mimicry through amplification of IL-6 expression in glioma cells

Supplementary Materials

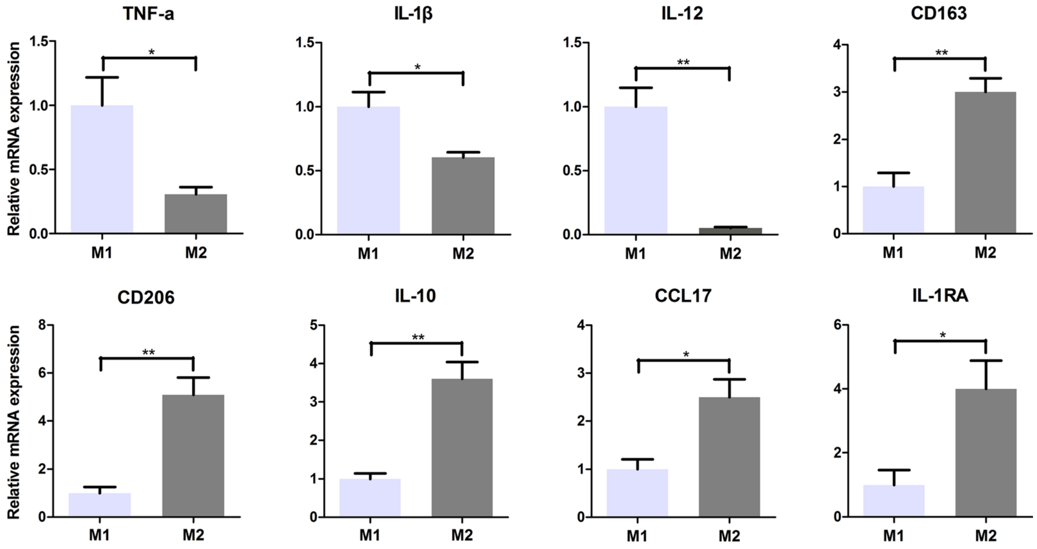

Supplementary Figure S1: M2-polarized macrophages were generated by treating THP-1 cells with PMA for 24 h and polarizing them with 20 ng/ml IL-4 and 20 ng/ml IL-13 for 18 h (added 6 h after PMA). M1-polarized macrophages were obtained from THP-1 cells by treating them with PMA for 24 h and polarizing them with 20 ng/ml IFN-γ and 100 ng/ml LPS for 18 h (added 6 h after PMA). qRT-PCR assay showed that transcription of TNF-a, IL-1β and IL-12 was increased in M1 macropahges compared to M2 macrophages, and transcription of CD163, CD206, IL-10, CCL17 and IL-1RA was increased in M2 macropahges compared to M1 macrophages. Each bar represents the mean ± SEM (n = 3, *P < 0.05, **P < 0.01).

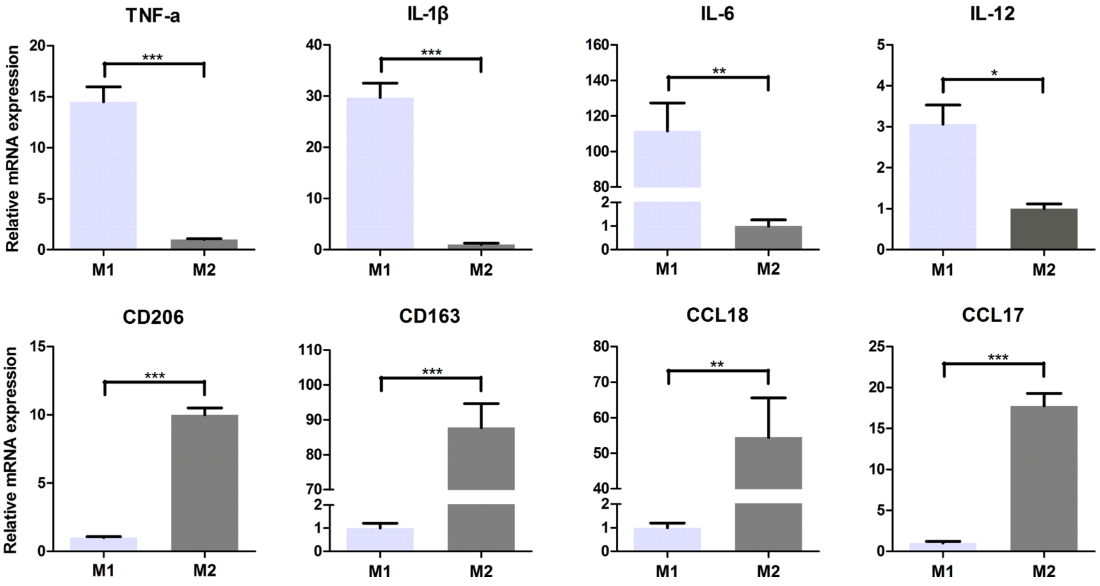

Supplementary Figure S2: Monocyte-derived M2 macrophages were obtained from human peripheral blood CD14⁺ monocytes treated with 100 ng/ml M-CSF for 5 days, followed by 20 ng/ml IL-4 and 20 ng/ml IL-13 for 3 days. Monocyte-derived M1 macrophages were obtained from human peripheral blood CD14⁺ monocytes treated with 50 ng/ml GM-CSF for 5 days, followed by 100 ng/ml LPS and 20 ng/ml IFN-γ for 3 days. qRT-PCR assay showed that transcription of TNF-a, IL-1β, IL-6 and increased in M2 macropahges compared to M1 macrophages. Each bar represents the mean ± SEM (n = 3, *P < 0.05; **P < 0.01; ***P < 0.001).

Supplementary Table S1: Primer sequences used in qRT-PCR

Gene name		Primer sequences	Species
CCL17	Forward	5′-AGAGCCACAGTGAGGGAGAT-3′	Human
	Reverse	5′-TTAATCTGGGCCCTTTGTGC-3′	
MMP2	Forward	5′-CAAGTTTCCATTCCGCTTC-3′	Human
	Reverse	5′-GTTCCCACCAACAGTGGACA-3′	
MMP9	Forward	5′-TTGACAGCGACAAGAAGTGGG-3′	Human
	Reverse	5′-GCCATTCACGTCGTCCTTAT-3′	
IL-1β	Forward	5′-TGATGGCTTATTACAGTGGCAATG-3′	Human
	Reverse	5′-GTAGTGGTGGTCGGAGATTCG-3′	
IL-10	Forward	5′-TCAAGGCGCATGTGAACTCC-3′	Human
	Reverse	5′-GATGTCAAACTCACTCATGGCT-3′	
IL-12	Forward	5′-GGAAGCACGGCAGCAGAATA-3′	Human
	Reverse	5′-AACTTGAGGGAGAAGTAGGAATGG-3′	
MMP14	Forward	5′-CCCCGAAGCCTGGCTACA-3′	Human
	Reverse	5′-GCATCAGCTTTGCCTGTTACT-3′	
IL-6	Forward	5′-CACACAGACAGCCACTCACC-3′	Human
	Reverse	5′-GCTCTGGCTTGTTCCTCACT-3′	
EPHA2	Forward	5′-ACTACGGCACCAACTTCCAG-3′	Human
	Reverse	5′-GTAGAAGCCTTTGCGGGTGA-3′	
VE-cadherin	Forward	5′-GTTCACGCATCGGTTGTTCAA-3′	Human
	Reverse	5′-CGCTTCCACCACGATCTCATA-3′	
LAMC2	Forward	5′-CAAAGGTTCTCTTAGTGCTCGAT-3′	Human
	Reverse	5′-CACTTGGAGTCTAGCAGTCTCT-3′	
nestin	Forward	5′-CTGCTACCCTTGAGACACCTG-3′	Human
	Reverse	5′-GGGCTCTGATCTCTGCATCTAC-3′	
Vimentin	Forward	5′-TGCCGTTGAAGCTGCTAACTA-3′	Human
	Reverse	5′-CCAGAGGGAGTGAATCCAGATTA-3′	
CCL18	Forward	5′-TCTATACCTCCTGGCAGATTC-3′	Human
	Reverse	5′-TTTCTGGACCCACTTCTTATTG-3′	
N-cadherin	Forward	5′-AGCCAACCTTAACTGAGGAGT-3′	Human
	Reverse	5′-GGCAAGTTGATTGGAGGGATG-3′	
COX-2	Forward	5′-TAAGTGCGATTGTACCCGGAC-3′	Human
	Reverse	5′-TTTGTAGCCATAGTCAGCATTGT-3′	
MMP-12	Forward	5′-GGAATCCTAGCCCATGCTTTT-3′	Human
	Reverse	5′-CATTACGGCCTTTGGATCACT-3′	
MMP-25	Forward	5′-CAGAACGGGAAGACCTACCTG-3′	Human
	Reverse	5′-GTTGCTGACGGTGACATCGT-3′	
TNF-a	Forward	5′-CCTCTCTCTAATCAGCCCTCTG-3′	Human
	Reverse	5′-GAGGACCTGGGAGTAGATGAG-3′	
CD163	Forward	5′-GACGCATTTGGATGGATCATGT-3′	Human
	Reverse	5′-CCCACCGTCCTTGGAATTTGA-3′	
CD206	Forward	5′-GGGTTGCTATCACTCTCTATGC-3′	Human
	Reverse	5′-TTTCTTGTCTGTTGCCGTAGTT-3′	
IL-1RA	Forward	5′-CATTGAGCCTCATGCTCTGTT-3′	Human
	Reverse	5′-CGCTGTCTGAGCGGATGAA-3′	
GAPDH	Forward	5′-GGTGGTCTCCTCTGACTTCAACAG-3′	Human
	Reverse	5′-GTTGCTGTAGCCAAATTCGTTGT-3′	
